# Supplementary material for: Optimization of Gefitinib-Loaded Nanostructured Lipid Carrier as a Biomedical Tool in the Treatment of Metastatic Lung Cancer
Source: Molecules. 2023 Jan 3;28(1):448. doi: 10.3390/molecules28010448 (PMC9823586; doi:10.3390/molecules28010448)
Supplement: Supplementary file 1 [file molecules-28-00448-s001.zip › molecules-2049003-supplementary.pdf]

**Table S1:** list of abbreviations

| Abbreviation | Meaning |
|---|---|
| **DOE** | Design of Experiments |
| **GEF** | Gefitinib |
| **LCFA** | Long-chain fatty acid; oleic acid |
| **LCM** | Long-chain monoglyceride; glycerol monolinoleate |
| **LCT** | Long-chain triglyceride; soybean |
| **NLC** | Nanostructured lipid carrier |
| **PDI** | Polydispersity index |
| **PS** | Particle size |
| **SA** | Stearic acid |
| **SL/LO** | Solid lipid: liquid oil ratio |
| **SLN** | Solid lipid nanoparticles |
| **ZP** | Zeta potential |
